# Automated CT-based quantification of knee osteophytes for robotic-assisted total knee arthroplasty: a multi-cohort study

**DOI:** 10.1007/s11701-026-04015-y

**Published:** 2026-10-06

**Authors:** Zhiqian Zheng, Do Weon Lee, Sewon Jeon, Hong Yeol Yang, Jong Keun Seon, Ho-Jung Jung, Joong Il Kim, Byung Sun Choi, Hyuk-Soo Han, Du Hyun Ro

**Affiliations:** 1 Department of Orthopedic Surgery, Seoul National University Hospital, Seoul, Republic of Korea; 2 Department of Orthopedic Surgery, Seoul National University College of Medicine, Seoul, Republic of Korea; 3 CONNECTEVE Co., Ltd, Seoul, Republic of Korea; 4 Department of Orthopedic Surgery, Chonnam National University Hwasun Hospital, Hwasun, Republic of Korea; 5 Department of Orthopedic Surgery, Dongguk University Ilsan Hospital, Goyang-si, South Korea; 6 Department of Orthopedic Surgery, Kangnam Sacred Heart Hospital, Hallym University College of Medicine, Seoul, Korea

**Keywords:** Computed tomography, Deep learning, Image segmentation, Osteophyte, Robotic arthroplasty, Total knee arthroplasty

## Abstract

Preoperative computed tomography (CT) is routinely acquired for planning robotic-assisted total knee arthroplasty (TKA), yet osteophyte burden is not quantitatively characterized. We developed automated CT-based osteophyte quantification and evaluated it against blinded de novo expert-consensus annotations and an intraoperative accessible resected osteophyte volume reference. A human-in-the-loop 3-dimensional U-Net was developed using 387 knee CT scans. The frozen model was evaluated against blinded de novo expert-consensus annotations in two independent external cohorts (100 knees each). Spatial and volume agreement were assessed using Dice coefficient, surface Dice at 1-mm tolerance, 95th-percentile Hausdorff distance (HD95), intraclass correlation coefficient (ICC), mean absolute error, and Bland–Altman analysis. In a prospective cohort of 66 robotic-assisted TKAs, CT-derived total osteophyte volume was compared with the water-displacement volume of accessible osteophytes that were resected and recovered intraoperatively (accessible resected osteophyte volume reference). In the external cohorts, median total Dice was 0.913 and 0.909, surface Dice was 0.944 and 0.945, HD95 was 1.16 and 1.09 mm, and volume ICC was 0.977 and 0.972, respectively. Automated estimates showed conservative volume bias relative to expert consensus (− 0.499 and − 0.365 cc). In the surgical cohort, automated CT-derived volume showed cohort-level agreement with the accessible resected osteophyte volume reference (ICC, 0.876; mean absolute error, 1.224 cc; mean difference, + 0.401 cc; 95% limits of agreement, − 2.785 to + 3.586 cc). Automated CT-based osteophyte quantification showed consistent spatial and volumetric agreement with blinded de novo expert-consensus annotations across two external cohorts and cohort-level agreement with the accessible resected osteophyte volume reference. This scalable method provides a preoperative measure of osteophyte burden for arthroplasty research.

**Supplementary Information:**

The online version contains supplementary material available at https://doi.org/10.1007/s11701-026-04015-y.

## Introduction

Knee osteoarthritis (OA) is a leading cause of musculoskeletal disability worldwide [1]. Osteophytes are a characteristic structural feature of OA and are routinely encountered during total knee arthroplasty (TKA) [2]. However, conventional radiographs provide only a two-dimensional and qualitative representation of osteophyte formation. They cannot quantify three-dimensional osteophyte burden or consistently distinguish femoral from tibial involvement. This limitation is particularly relevant in robotic-assisted TKA, in which preoperative computed tomography (CT) is acquired for three-dimensional planning and registration [3].

CT enables detailed three-dimensional assessment of osseous morphology and has been used for manual or semi-automated osteophyte quantification in knee OA [4–6]. However, these approaches are not scalable for large clinical or research cohorts. Recent deep learning studies have assessed osteophyte detection on radiographs and magnetic resonance imaging, but automated three-dimensional CT quantification of knee osteophytes remains insufficiently validated [7]. In particular, volumetric agreement alone is inadequate for a segmentation task because anatomically discordant masks may yield similar total volumes. Robust validation should therefore assess both spatial localization and volume agreement against independently generated imaging-based reference annotations.

We developed an automated CT-based method for total and compartment-specific knee osteophyte quantification in the robotic-assisted TKA setting. The primary aim was to evaluate the frozen model in two independent external cohorts using blinded de novo expert-consensus annotations, with spatial and volumetric agreement assessed for total, femoral, and tibial osteophytes. In a separate prospective surgical cohort, we compared automated CT-derived total osteophyte volume with the aggregate water-displacement volume of accessible osteophytes resected intraoperatively. This measurement was considered a clinically relevant physical reference for aggregate accessible osteophyte burden, rather than a complete reference for all CT-detectable osteophytes. Secondary exploratory analyses examined associations of CT-derived osteophyte burden with radiographic joint-space width, Kellgren–Lawrence grade, and coronal alignment. Establishing a validated and scalable preoperative measure of osteophyte burden provides a foundation for future studies of its potential role in robotic TKA planning, soft-tissue balancing, and clinical outcomes.

## Materials and methods

### Study design and ethical approval

This multi-cohort study was approved by the Seoul National University Hospital Institutional Review Board (approval no. 2506-043-1647) and conducted in accordance with the Declaration of Helsinki. Written informed consent was obtained from participants in the prospective surgical cohort; the requirement for informed consent was waived for the retrospective cohorts.

**Fig. 1:**
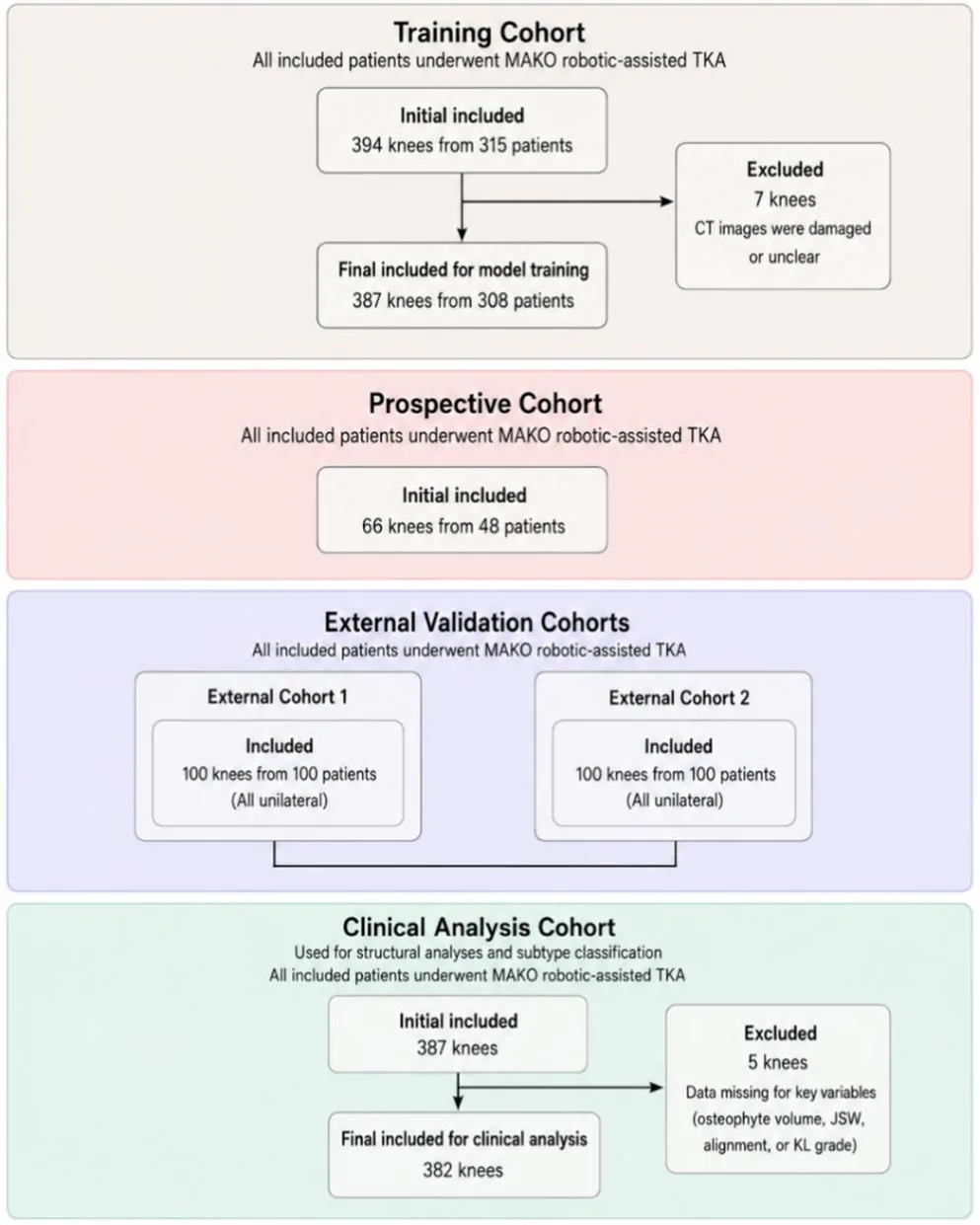
Patient enrollment and cohort selection. The training cohort included 394 screened knees from 315 patients; 7 CT scans with inadequate image quality were excluded, leaving 387 knees from 308 patients for model development. The prospective surgical cohort included 66 knees from 48 patients. Each external cohort included 100 unilateral knees. After exclusion of 5 knees with incomplete data, 382 training-cohort knees entered exploratory structural analyses

Four cohorts of patients undergoing Mako robotic-assisted total knee arthroplasty at three institutions were included (Table 1): a training cohort (*n* = 387) for human-in-the-loop model development; a prospective surgical cohort (*n* = 66) for comparison with the intraoperative resection-volume reference; and two independent external cohorts (*n* = 100 each) for evaluation against blinded de novo expert-consensus annotations. The frozen model was not retrained or otherwise adapted using the prospective or external cohorts. Secondary exploratory structural analyses included 382 training-cohort knees with complete osteophyte-volume, radiographic, and alignment data (Fig. 1). Eligible patients had a preoperative knee CT; scans with inadequate image quality were excluded.

**Table 1:** Design and composition of the four study cohorts

Characteristic	Training cohort	Prospective cohort	External cohort 1	External cohort 2
Purpose	Model training & clinical analysis	Agreement with intraoperative resection volume	External validation	External validation
Design	Retrospective	Prospective	Retrospective	Retrospective
Institution	Institution A	Institution A	Institution B	Institution C
n	387 (382 with complete radiographic data)	66	100	100
Study period	Jan 2022–Mar 2025	Apr 2025–Apr 2026	Jan 2023–Oct 2025	Sep 2022–Dec 2025
Procedure	Mako robotic-assisted TKA	Mako robotic-assisted TKA	Mako robotic-assisted TKA	Mako robotic-assisted TKA

TKA, total knee arthroplasty. Institution labels are anonymized for peer review. The prospective and external cohorts were not used for model development

### CT acquisition and reconstruction

All preoperative noncontrast CT examinations were acquired using a standardized robotic TKA planning protocol for lower-extremity alignment and three-dimensional planning on a Philips IQon Spectral CT system (Philips Healthcare, Best, the Netherlands). Acquisition parameters were 120 kVp and 196 mA. Images were reconstructed using a YC kernel with a 250-mm reconstruction diameter, 0.67-mm slice thickness and interval, and an in-plane pixel spacing of 0.488 × 0.488 mm.

### CT annotation and human-in-the-loop segmentation model development

In the model-development cohort, two orthopaedic surgeons used 3D Slicer (version 5.6.1; https://www.slicer.org) to delineate the distal femur, proximal tibia, and osteophytes on CT, with cross-review for consistency. Osteophytes were defined as osseous outgrowths distinct from the parent-bone cortex.

**Fig. 2:**
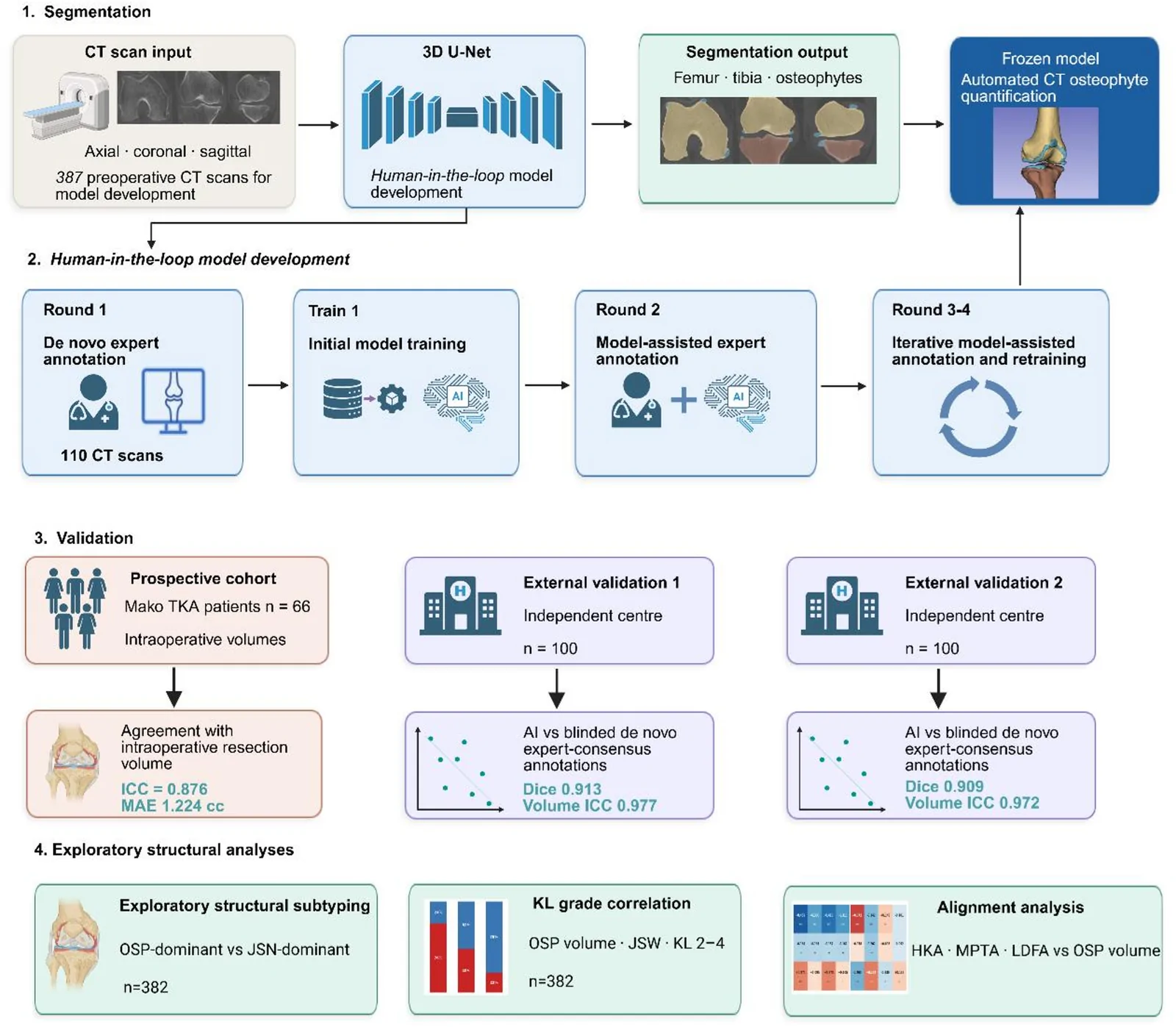
Study workflow. (**1**) A human-in-the-loop 3D U-Net was developed using 387 preoperative knee CT scans to segment the distal femur, proximal tibia, and osteophytes. (**2**) The initial model was trained on 110 de novo expert-annotated scans and iteratively retrained using model-assisted annotations. (**3**) The frozen model was evaluated against the intraoperative resection-volume reference in 66 robotic-assisted TKAs and against blinded de novo expert-consensus annotations in two external cohorts (100 knees each). (**4**) Exploratory analyses in 382 training-cohort knees examined associations with joint-space width, Kellgren–Lawrence grade, and coronal alignment. AI, artificial intelligence; CT, computed tomography; JSW, joint-space width; OSP, osteophyte; TKA, total knee arthroplasty

A human-in-the-loop strategy was used to construct the model-development dataset. An initial 3-dimensional U-Net was trained using 110 de novo expert-annotated CT scans. In three subsequent rounds, model-generated segmentations of newly selected scans underwent expert review and correction of missed osteophytes and false-positive regions before retraining. This expanded the cumulative dataset to 210, 310, and 387 scans. Within each round, the annotated scans were divided into development and internal-validation subsets (100/10, 190/20, 280/30, and 347/40 scans, respectively), and the checkpoint with the lowest internal-validation loss was selected. Internal-validation osteophyte Dice coefficients were 0.69, 0.78, 0.79, and 0.827 across the four rounds. After the final round, the model was frozen and was not retrained, adapted, or used for threshold selection in the prospective surgical or external validation cohorts (Fig. 2). Detailed model architecture, preprocessing, augmentation, training, and inference procedures are provided in Online Resource 1 (Supplementary methods).

### Compartmental segmentation and volume computation

AI-generated femoral, tibial, and osteophyte masks were post-processed using a custom Python pipeline (version 3.12.6). A sagittal plane through the femoral intercondylar-notch midpoint or tibial spine divided each parent bone and its associated osteophytes into medial and lateral compartments, yielding femur medial, femur lateral, tibia medial, and tibia lateral osteophyte volumes. Volumes were calculated by voxel summation in mm³. For agreement with intraoperative water-displacement measurements, total osteophyte volumes were converted to cc (1 cc = 1000 mm³).

### CT-based coronal alignment measurement

Coronal alignment was measured from full-length lower-extremity images obtained during the same preoperative CT examination used for osteophyte segmentation; no additional CT examination was performed. Femoral head centre, medial and lateral femoral epicondyles, proximal tibial centre, and medial and lateral malleoli were manually identified using a custom 3D Slicer pipeline (Python 3.12.6). The distal femur centre, ankle centre, and articular reference points were automatically derived from these landmarks and the segmented bone surfaces. The femoral mechanical axis connected the femoral head centre and distal femur centre, and the tibial mechanical axis connected the proximal tibial centre and ankle centre. CT-HKA, CT-MPTA, and CT-LDFA were calculated directly in three-dimensional space using signed angles. CT-HKA values below and above 180° indicated varus and valgus alignment, respectively. Landmark definitions and angle construction are shown in Online Resource 1 (Supplementary Fig. S1).

### Radiographic assessment

Kellgren–Lawrence (KL) grade and joint-space width (JSW) were automatically derived from standing knee radiographs using a commercial AI radiographic-analysis platform (CONNEVO KOA, version 1.0.0; CONNECTEVE Co., Ltd.) [8]. For each knee, the platform generated KL grade and four JSW measurements: anteroposterior (AP) medial, AP lateral, posteroanterior (PA) medial, and PA lateral JSW (mm). Knees with failed or missing measurements were excluded from the corresponding exploratory analyses.

### Agreement with intraoperative resection volume

**Fig. 3:**
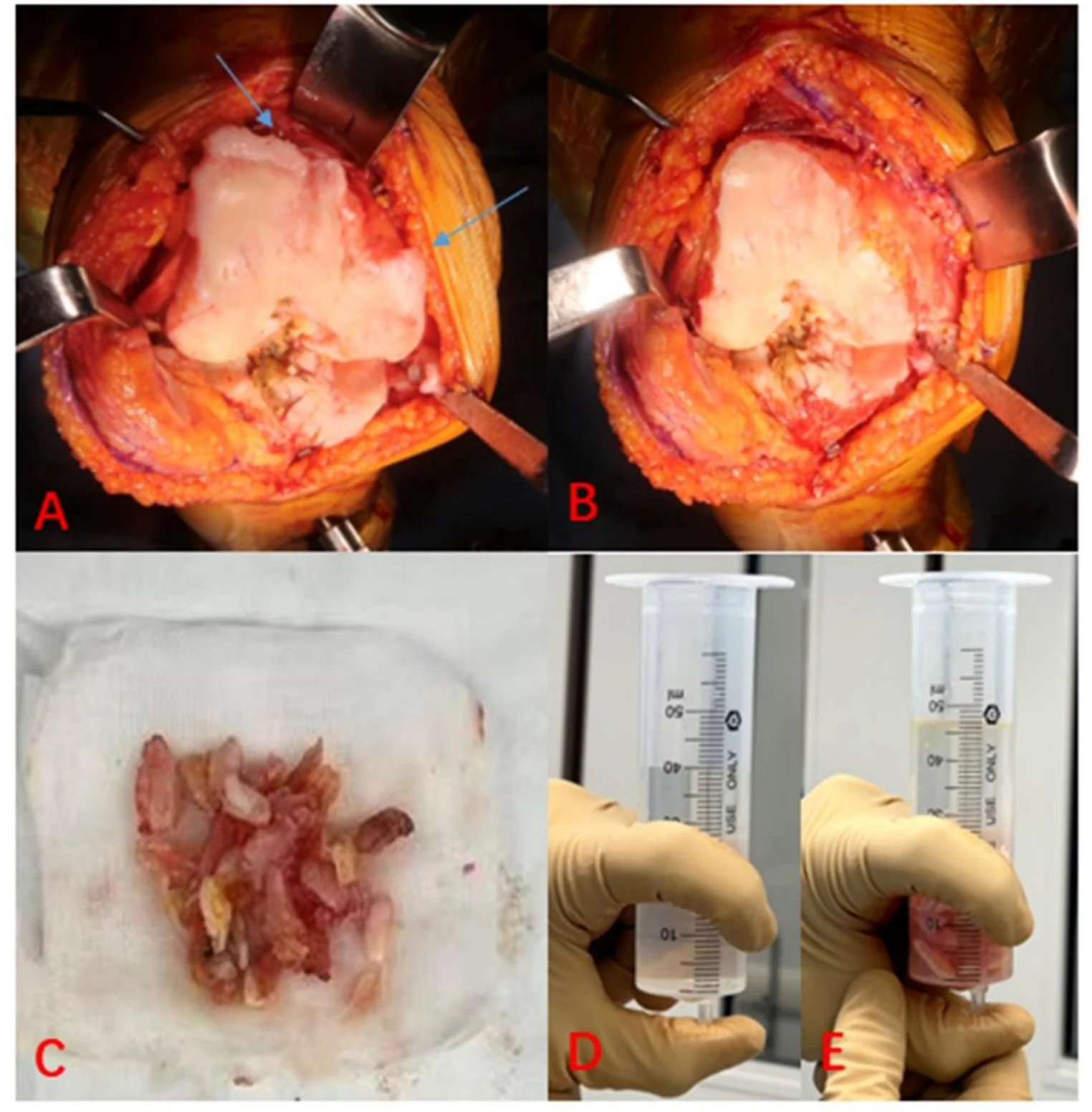
Intraoperative resection and volumetric measurement of knee osteophytes. **(A)** Intraoperative view before resection; blue arrows indicate marginal osteophytes at the femoral condyle. **(B)** The same view after systematic resection of accessible osteophytes. **(C)** Resected osteophyte specimens collected together after removal, with attached soft tissue excluded. **(D)** A 50-mL syringe prefilled with 40 mL of water before specimen immersion. **(E)** The same syringe after addition of the resected specimens; the displaced volume represents the aggregate volume of accessible osteophytes resected intraoperatively and was read once from the syringe scale to an approximate resolution of 1 cc (1 mL = 1000 mm³)

In 66 Mako robotic-assisted TKAs performed by a single surgeon, automated CT-derived total osteophyte volume was compared with the aggregate volume of accessible osteophytes resected intraoperatively (Fig. 3). CT was acquired within 1 week before surgery. During standard exposure, accessible femoral and tibial osteophytes were resected, whereas posterior osteophytes not reached during exposure could remain in situ. Fresh resected specimens, with attached soft tissue excluded, were measured once by water displacement in a 50-mL syringe prefilled with 40 mL of water (1 mL = 1000 mm³). The operating surgeon had no access to, and was unaware of, the automated CT-derived osteophyte volume at the time of surgery and water-displacement measurement. Duplicate measurements and formal calibration or repeatability assessment of the syringe-based procedure were not performed. Bilateral knees were collected and measured separately.

The intraoperative resection-volume measurement served as a clinically relevant physical reference for aggregate accessible osteophyte burden; it was not considered a complete reference for all CT-detectable osteophytes.

### External validation

The frozen model was evaluated in two independent external cohorts (100 knees each). Each external cohort comprised the first 100 consecutive eligible unilateral knees from patients undergoing Mako robotic-assisted TKA during the respective study period. No additional case selection was performed. For every knee, two orthopaedic surgeons, each with more than 5 years of relevant clinical experience, independently performed de novo annotations of femoral, tibial, and total osteophytes from the original CT images while blinded to the AI-generated segmentations. Both surgeons received standardized training in the predefined annotation protocol and used 3D Slicer (version 5.6.1). Discrepancies between the independent annotations were resolved through joint review to generate a single expert-consensus reference annotation. Osteophytes were defined as osseous outgrowths distinct from the parent-bone cortex, consistent with the model-development annotation protocol. For expert annotation, the parent-bone origin of each osteophyte was determined from direct cortical continuity with the femur or tibia on multiplanar CT images. Three-dimensional renderings were used during consensus review when parent-bone attachment required further confirmation. AI and consensus masks were compared in the common CT coordinate space. Total osteophytes were assigned to the femur or tibia according to the nearest parent-bone mask, using the identical rule for AI and consensus annotations.

### Exploratory structural subtyping

Among 382 training-cohort knees, total osteophyte volume and AP medial joint-space width (JSW) were standardized to cohort z-scores. Knees with z_OSP + z_JSW > 0 were classified as osteophyte-dominant and all others as joint-space-narrowing-dominant (Online Resource 1, Supplementary Fig. S2B). As lower JSW indicates greater narrowing, this rule reflects the relative predominance of osteophyte burden over joint-space narrowing. This exploratory classification was not intended to define validated OA phenotypes.

### Statistical analysis

Continuous variables are reported as mean ± SD and categorical variables as n (%). Normality was assessed using the Shapiro–Wilk test. Because osteophyte volumes were non-normally distributed, group comparisons used Mann–Whitney U or Kruskal–Wallis tests with Dunn post-hoc testing; joint-space-width (JSW) comparisons between exploratory structural categories were Bonferroni-corrected. Exploratory associations were assessed using Spearman correlation.

Compartment-specific osteophyte volumes were modelled using multivariable linear regression including CT-HKA, CT-MPTA, CT-LDFA, age, and sex. Analyses were restricted to KL grade 2–4 knees, used patient-ID cluster-robust standard errors, and were repeated after log1p transformation. Agreement with the intraoperative resection-volume reference was assessed using ICC(2,1), percentile-bootstrap 95% confidence intervals (2000 resamples), MAE, mean difference, and Bland–Altman 95% limits of agreement (LoA). External validation used Dice coefficient, surface Dice at a 1-mm tolerance, and HD95, reported as median (IQR), together with ICC(2,1), MAE, bias, and Bland–Altman LoA for total, femoral, and tibial volumes. Analyses used Python 3.12.6; *p* < 0.05 was considered statistically significant.

## Results

### Study cohorts

The exploratory structural analysis cohort comprised 382 knees with complete radiographic and alignment data (KL grade 2–4; mean age, 74.1 ± 6.6 years; 78.5% women). The prospective surgical cohort included 66 knees (mean age, 70.1 ± 5.9 years; 84.8% women). External cohorts 1 and 2 included 100 knees each (mean age, 72.2 ± 6.1 and 70.4 ± 6.3 years; 81.0% and 90.2% women, respectively). Cohort design and composition are summarized in Table 1.

### Human-in-the-loop model development

The human-in-the-loop strategy expanded the model-development dataset from 110 de novo annotated CT scans to 387 scans through three subsequent rounds of model-assisted expert annotation and retraining (Fig. 2). The resulting model was frozen and evaluated without further adaptation in the prospective surgical cohort and two independent external validation cohorts.

### Agreement with intraoperative resection volume

Among 66 prospective robotic-assisted TKA knees, mean automated CT-derived total osteophyte volume was 5.666 ± 3.558 cc, compared with 5.265 ± 3.073 cc for the intraoperative resection-volume reference. Automated CT-derived volume showed cohort-level agreement with the intraoperative resection-volume reference (ICC(2,1), 0.876; 95% CI, 0.793–0.924; Table 2), with an MAE of 1.224 cc. Bland–Altman analysis showed that automated CT estimates were higher by a mean of 0.401 cc (AI minus intraoperative resection volume), with 95% limits of agreement of − 2.785 to + 3.586 cc.

**Table 2:** Agreement between automated CT-derived total osteophyte volume and the intraoperative accessible resected osteophyte volume reference in 66 robotic-assisted total knee arthroplasties

Comparison	ICC(2,1) (95% CI)	MAE (cc)	Mean difference (cc)	95% LoA (cc)
Automated CT-derived volume vs. intraoperative resection-volume reference	0.876 (0.793–0.924)	1.224	+ 0.401	−2.785 to + 3.586

Values are based on automated CT-derived total osteophyte volume minus the intraoperative resection-volume reference; thus, a positive mean difference indicates a higher automated CT-derived volume. The accessible resected osteophyte volume reference was measured by water displacement of osteophytes that were accessible, resected, and recovered during surgery; it does not represent all CT-detectable osteophytes. CI, confidence interval; ICC, intraclass correlation coefficient; LoA, limits of agreement; MAE, mean absolute error

### External validation

All 200 external knees were successfully evaluated against blinded de novo expert-consensus annotations (Table 3). In external cohort 1, total osteophyte segmentation achieved a median Dice coefficient of 0.913 (IQR, 0.833–0.945), surface Dice at a 1-mm tolerance of 0.944 (IQR, 0.903–0.961), and HD95 of 1.16 mm (IQR, 0.79–1.80). Total-volume ICC(2,1) was 0.977 (95% CI, 0.951–0.991). External cohort 2 showed comparable performance, with a Dice coefficient of 0.909 (IQR, 0.840–0.945), surface Dice of 0.945 (IQR, 0.906–0.970), HD95 of 1.09 mm (IQR, 0.62–2.00), and total-volume ICC(2,1) of 0.972 (95% CI, 0.961–0.983). Femoral and tibial volume ICCs were 0.987 and 0.934 in cohort 1 and 0.972 and 0.948 in cohort 2, respectively. In both cohorts, AI estimates were conservatively lower than expert-consensus volumes, with total-volume biases of − 0.499 and − 0.365 cc, respectively. Signed-error distributions and total-volume Bland–Altman plots are shown in Online Resource 1 (Supplementary Fig. S3). All total-volume AI-minus-expert-consensus differences were negative in external cohort 1 and were negative or zero in external cohort 2, explaining why MAE closely approximated the absolute mean bias in both cohorts. Representative cases illustrating posterior osteophyte undersegmentation and the effect of very low osteophyte volume on overlap metrics are provided in Online Resource 1 (Supplementary Fig. S4).

**Table 3:** External validation of automated CT-based osteophyte segmentation against blinded de novo expert-consensus annotations in two independent cohorts

	External cohort 1 (*n* = 100)	External cohort 2 (*n* = 100)
*Spatial agreement: total OSP*		
Dice, median (IQR)	0.913 (0.833–0.945)	0.909 (0.840–0.945)
Surface Dice at 1 mm, median (IQR)	0.944 (0.903–0.961)	0.945 (0.906–0.970)
HD95, mm, median (IQR)	1.16 (0.79–1.80)	1.09 (0.62–2.00)
*Spatial agreement: femoral OSP*		
Dice, median (IQR)	0.936 (0.861–0.962)	0.917 (0.850–0.961)
Surface Dice at 1 mm, median (IQR)	0.956 (0.932–0.976)	0.955 (0.907–0.977)
HD95, mm, median (IQR)	0.93 (0.62–1.41)	0.96 (0.41–1.72)
*Spatial agreement: tibial OSP*		
Dice, median (IQR)	0.856 (0.736–0.915)	0.906 (0.802–0.974)
Surface Dice at 1 mm, median (IQR)	0.909 (0.846–0.950)	0.943 (0.891–0.989)
HD95, mm, median (IQR)	1.46 (1.05–2.48)	1.13 (0.08–2.00)
*Volume agreement: total OSP*		
ICC(2,1) (95% CI)	0.977 (0.951–0.991)	0.972 (0.961–0.983)
MAE, cc	0.499	0.365
Bias, cc	− 0.499	− 0.365
95% LoA, cc	− 1.684 to + 0.686	− 1.207 to + 0.477
*Volume agreement: femoral OSP*		
ICC(2,1) (95% CI)	0.987 (0.965–0.996)	0.972 (0.957–0.988)
MAE, cc	0.287	0.259
Bias, cc	− 0.287	− 0.259
95% LoA, cc	− 1.016 to + 0.442	− 0.977 to + 0.459
*Volume agreement: tibial OSP*		
ICC(2,1) (95% CI)	0.934 (0.902–0.967)	0.948 (0.900–0.973)
MAE, cc	0.212	0.106
Bias, cc	− 0.212	− 0.106
95% LoA, cc	− 0.773 to + 0.348	− 0.381 to + 0.170

Spatial metrics are reported as median (interquartile range). Volume ICCs are ICC(2,1) with percentile-bootstrap 95% confidence intervals. Bias was calculated as AI-derived volume minus expert-consensus volume; negative values indicate lower AI-derived estimates. HD95, 95th-percentile Hausdorff distance; ICC, intraclass correlation coefficient; LoA, limits of agreement; MAE, mean absolute error; OSP, osteophyte

### Osteophyte volumes, JSW, and KL grade

Among 382 knees, osteophyte volumes were greatest in the femur medial compartment and lowest in the tibia lateral compartment, with right-skewed distributions in all four compartments (Online Resource 1,Supplementary Table S1). Volumes increased across KL grades in every compartment (Kruskal–Wallis *p* < 0.001). Total osteophyte volume correlated modestly with KL grade (Spearman ρ = 0.25), whereas KL grade correlated strongly with AP medial JSW (ρ = −0.70), weakly with PA medial JSW (ρ = − 0.21), and negligibly with lateral JSW measurements (Online Resource 1,Supplementary Table S1). Total osteophyte volume and AP medial JSW were weakly correlated (ρ = − 0.20, *p* < 0.001; Online Resource 1, Supplementary Fig. S2B).

### Exploratory structural subtyping

Of 382 knees, 163 (42.7%) were classified as osteophyte-dominant and 219 (57.3%) as joint-space-narrowing-dominant. The proportion in the joint-space-narrowing-dominant category increased across KL grades (24.2%, 52.1%, and 78.4% for KL grades 2, 3, and 4, respectively; Online Resource 1, Supplementary Fig. S2A). As defined, osteophyte-dominant knees had greater osteophyte volumes in all compartments and wider AP medial JSW (all *p* < 0.001). PA medial and lateral JSW measurements and CT-HKA did not differ between categories (Online Resource 1, Supplementary Fig. S2C).

### Alignment and compartment-specific osteophyte burden

CT-HKA correlated negatively with osteophyte volume in all compartments, most strongly in the femur medial compartment (ρ = − 0.46, *p* < 0.001; Online Resource 1, Supplementary Fig. S5A). In exploratory multivariable models adjusted for CT-MPTA, CT-LDFA, age, and sex using patient-ID cluster-robust standard errors, lower CT-HKA was associated with greater osteophyte volume in all compartments (all *p* ≤ 0.039; Online Resource 1, Supplementary Fig. S5B; Supplementary Table S2). CT-LDFA was independently associated only with tibia medial volume (*p* = 0.031), whereas CT-MPTA showed no consistent independent association.

## Discussion

We developed and externally validated an automated CT-based method for three-dimensional quantification of knee osteophytes in robotic-assisted TKA. The primary evidence was consistent agreement with blinded de novo expert-consensus annotations in two independent external cohorts. Median total-osteophyte Dice coefficients were 0.913 and 0.909, surface Dice values were 0.944 and 0.945, and total-volume ICCs were 0.977 and 0.972. In the prospective surgical cohort, automated CT-derived volume also showed cohort-level agreement with the aggregate volume of accessible osteophytes resected intraoperatively (ICC, 0.876; MAE, 1.224 cc). These findings support automated CT quantification as a scalable measure of preoperative osteophyte burden.

External validation assessed both spatial localization and volume estimation, because similar volumes may arise from anatomically discordant segmentations [9]. High surface Dice, low HD95, and consistently high femoral and tibial volume ICCs indicate agreement in both osteophyte location and parent-bone assignment. Automated estimates were modestly lower than expert-consensus volumes in both cohorts (bias, − 0.499 and − 0.365 cc). This reproducible direction suggests conservative segmentation of marginal osteophytes rather than unstable external performance. In knees with very low osteophyte volume, small boundary discrepancies can disproportionately affect overlap-based metrics; the high volume ICCs and low absolute errors indicate limited cohort-level influence.

The intraoperative comparison provides a clinically relevant physical reference that complements, rather than replaces, imaging-based validation. Water displacement measured osteophytes that were accessible, resected, and recovered during routine surgery, but not necessarily all CT-detectable osteophytes; posterior lesions may remain unexposed, unresected, or uncollected. Posterior osteophytes can influence extension and gap balance during TKA, supporting the distinction between total CT-detectable and accessible resected burden [10, 11]. A single syringe measurement with approximately 1 cc resolution and incomplete posterior recovery may have contributed to the + 0.401 cc mean difference and observed LoA. Thus, the surgical analysis supports cohort-level clinical relevance, whereas external validation provides the primary evidence for segmentation accuracy. These data do not establish sufficient individual-knee precision for automated intraoperative decision-making.

Because CT is already acquired for many robotic TKA workflows, automated quantification could provide standardized aggregate and bone-specific osteophyte measures without additional imaging or manual volumetry. The present findings establish the measurement validity of this preoperative CT-derived biomarker and provide a foundation for evaluating its clinical utility in robotic TKA. Whether osteophyte quantification improves surgical planning, soft-tissue balancing, intraoperative decision-making, or patient outcomes requires prospective evaluation. The exploratory analyses further suggest that CT-derived osteophyte burden and radiographic joint-space narrowing capture related but nonidentical structural features of OA; however, the derived structural categories were not linked to outcomes and should be considered hypothesis-generating rather than validated OA phenotypes.

Limitations include restriction to KL grade 2–4 knees undergoing robotic-assisted TKA. In addition, the intraoperative accessible resected osteophyte volume reference was obtained from a single water-displacement measurement using a 50-mL syringe with an approximate 1-cc reading resolution. Duplicate measurements and formal calibration or repeatability testing were not performed; therefore, the measurement uncertainty of this physical reference could not be quantified. Together with incomplete recovery of posterior osteophytes, these factors may have contributed to the observed CT-surgical limits of agreement and limit interpretation of this comparison to cohort-level agreement. Finally, the exploratory structural categories require independent longitudinal and clinical validation.

In conclusion, automated CT-based osteophyte quantification showed consistent spatial and volumetric agreement with blinded de novo expert-consensus annotations across two external cohorts and cohort-level agreement with the intraoperative accessible resected osteophyte volume reference. This method provides a scalable preoperative measure of osteophyte burden for arthroplasty research; its capacity to guide surgical decisions or improve outcomes requires prospective evaluation.

## Supplementary Information

Below is the link to the electronic supplementary material.

Supplementary Material 1

## Data Availability

The de-identified CT images and clinical data generated and analyzed in this study are not publicly available because of institutional and ethical restrictions governing patient data. Access may be considered upon reasonable request to the corresponding author, subject to approval by the institutional review board and applicable data-use requirements.
