# Supplementary material for: Automated CT-based quantification of knee osteophytes for robotic-assisted total knee arthroplasty: a multi-cohort study
Source: J Robot Surg. 2026 Oct 6;20(1):1066. doi: 10.1007/s11701-026-04015-y (PMC13642435; doi:10.1007/s11701-026-04015-y)
Supplement: Supplementary file 1 [file 11701_2026_4015_MOESM1_ESM.pdf]

Supplementary Methods

CT intensities were clipped between the 0.5th percentile and the maximum value of each volume and linearly rescaled to the range of 0 to 1. All volumes were resampled to a fixed voxel spacing of 0.488 × 0.488 × 0.6 mm. The model was trained using randomly extracted 160 × 128 × 128-voxel patches. Patch centres were sampled from foreground and background voxels at a 1:3 ratio, and each training batch comprised 14 patches sampled from a single volume.

To improve robustness to variation in patient positioning, anatomy, and image quality, spatial augmentations (random rotation, scaling, and flipping) and intensity augmentations (Gaussian noise, Gaussian smoothing, intensity scaling, and gamma correction) were applied during training. A combined Dice and cross-entropy loss was optimized using AdamW (weight decay, 0.05) for 150 epochs. The learning rate increased linearly to 0.001 during the first 5 epochs and then decreased to 0.00001 by cosine annealing. At inference, each CT volume was segmented using a sliding-window approach, and the predicted masks were resampled to the original CT resolution.

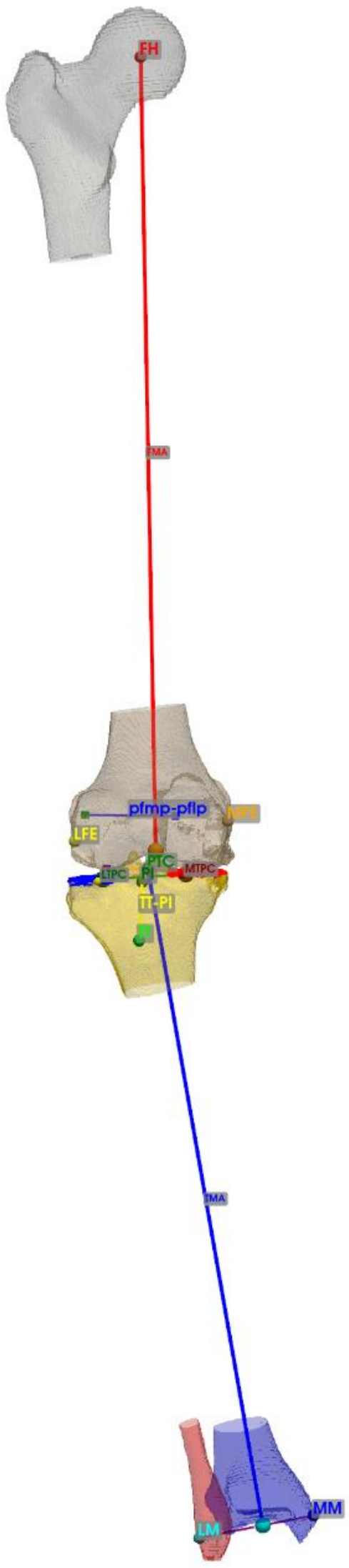

Supplementary Fig. S1. CT-based three-dimensional lower-limb alignment measurement. Three-dimensional rendering illustrates the landmarks, mechanical axes, and reference lines used by the custom CT-based alignment pipeline. The femoral mechanical axis (FMA; red) extends from the femoral head centre (FH) to the distal femur centre (DFC). The tibial mechanical axis (TMA; blue) extends from the proximal tibial centre (PTC) to the distal tibial centre (DTC). DTC was calculated as MM + 0.44 × (LM − MM), where MM and LM denote the medial and lateral malleoli, respectively. The distal femoral condylar line connects the distal femoral medial and lateral points (DFMP and DFLP), and the tibial plateau line connects the medial and lateral tibial plateau centres (MTPC and LTPC). CT-LDFA and CT-MPTA were calculated as signed three-dimensional angles between the corresponding

mechanical axis and distal femoral condylar or tibial plateau line, respectively. CT-HKA was calculated as 180° minus the signed three-dimensional angle between the femoral and tibial mechanical axes. MFE, medial femoral epicondyle; LFE, lateral femoral epicondyle; PI, posterior insertion landmark; TT, tibial tubercle.

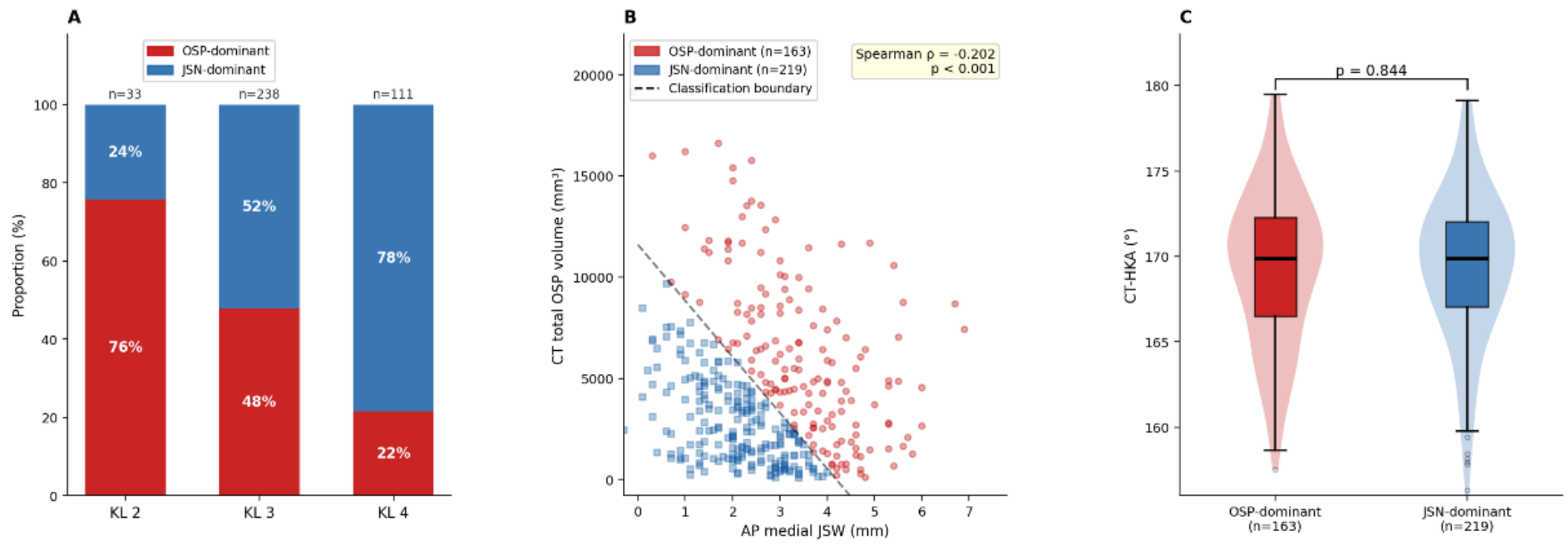

Supplementary Fig. S2. Exploratory structural subtyping in 382 model-development-cohort knees. (A) Category distribution by KL grade. (B) Standardized total osteophyte volume versus AP medial JSW; the dashed line indicates the classification boundary (z_OSP + z_JSW = 0). Knees above the line were osteophyte-dominant (n = 163) and those below were joint-space-narrowing-dominant (n = 219). (C) CT-HKA by category. Violin plots show distributions; boxes, interquartile ranges; horizontal lines, medians; whiskers, 1.5 × interquartile ranges. This classification is exploratory and does not represent validated OA phenotypes. CT-HKA, CT hip–knee–ankle angle; JSW, joint-space width; KL, Kellgren–Lawrence.

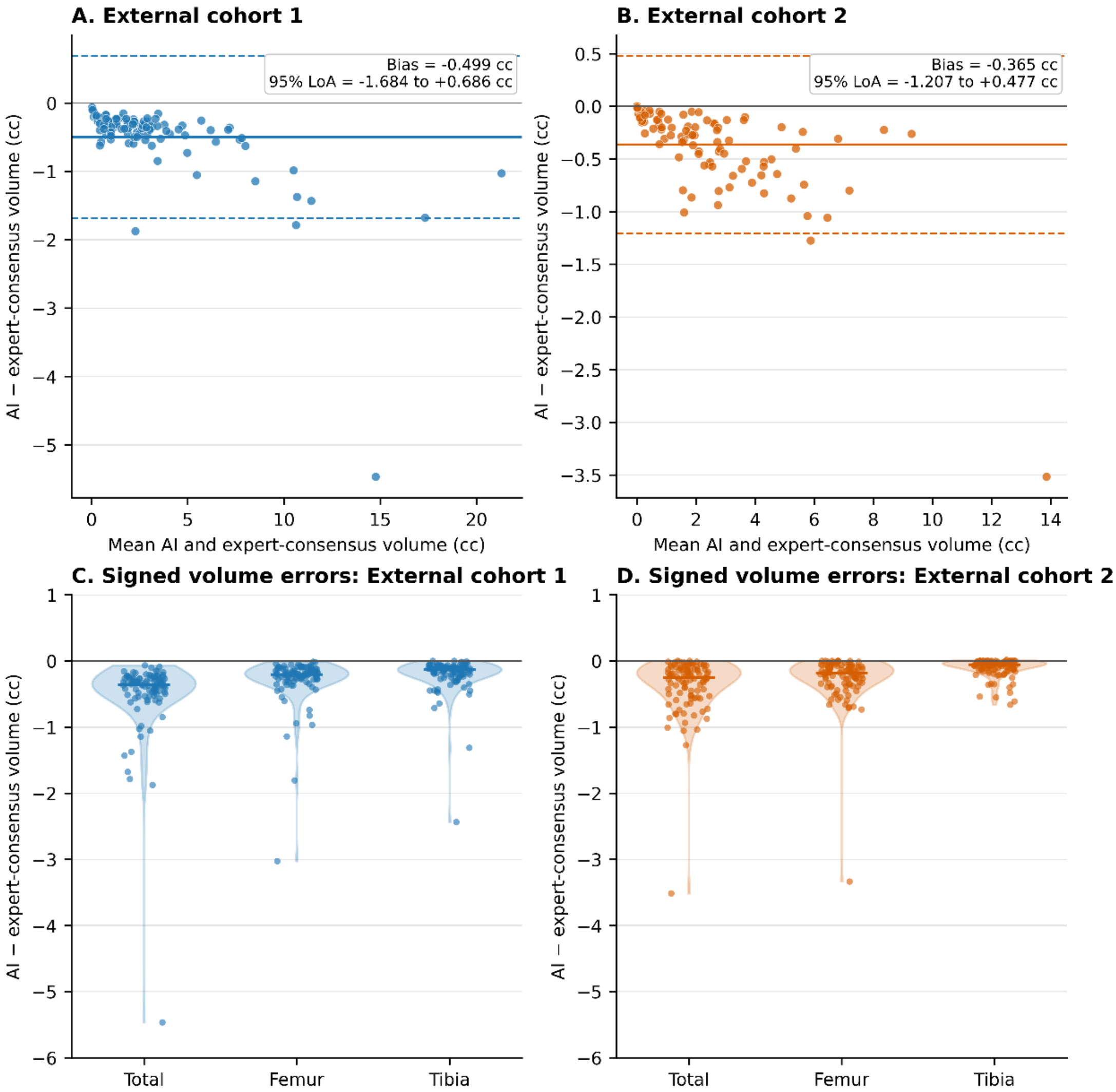

Supplementary Fig. S3. External volume-error distributions and Bland–Altman analyses. (A, B) Bland–Altman plots for total osteophyte volume in external cohorts 1 and 2, respectively. Solid colored lines indicate mean bias and dashed lines indicate the 95% limits of agreement; gray horizontal lines indicate zero difference. (C, D) Distributions of signed volume errors for total, femoral, and tibial osteophytes in external cohorts 1 and 2, respectively. Each point represents one knee; violin plots show distribution density and horizontal lines indicate the median. Signed error was calculated as automated volume minus blinded de novo expert-consensus volume; values below zero indicate lower automated estimates.

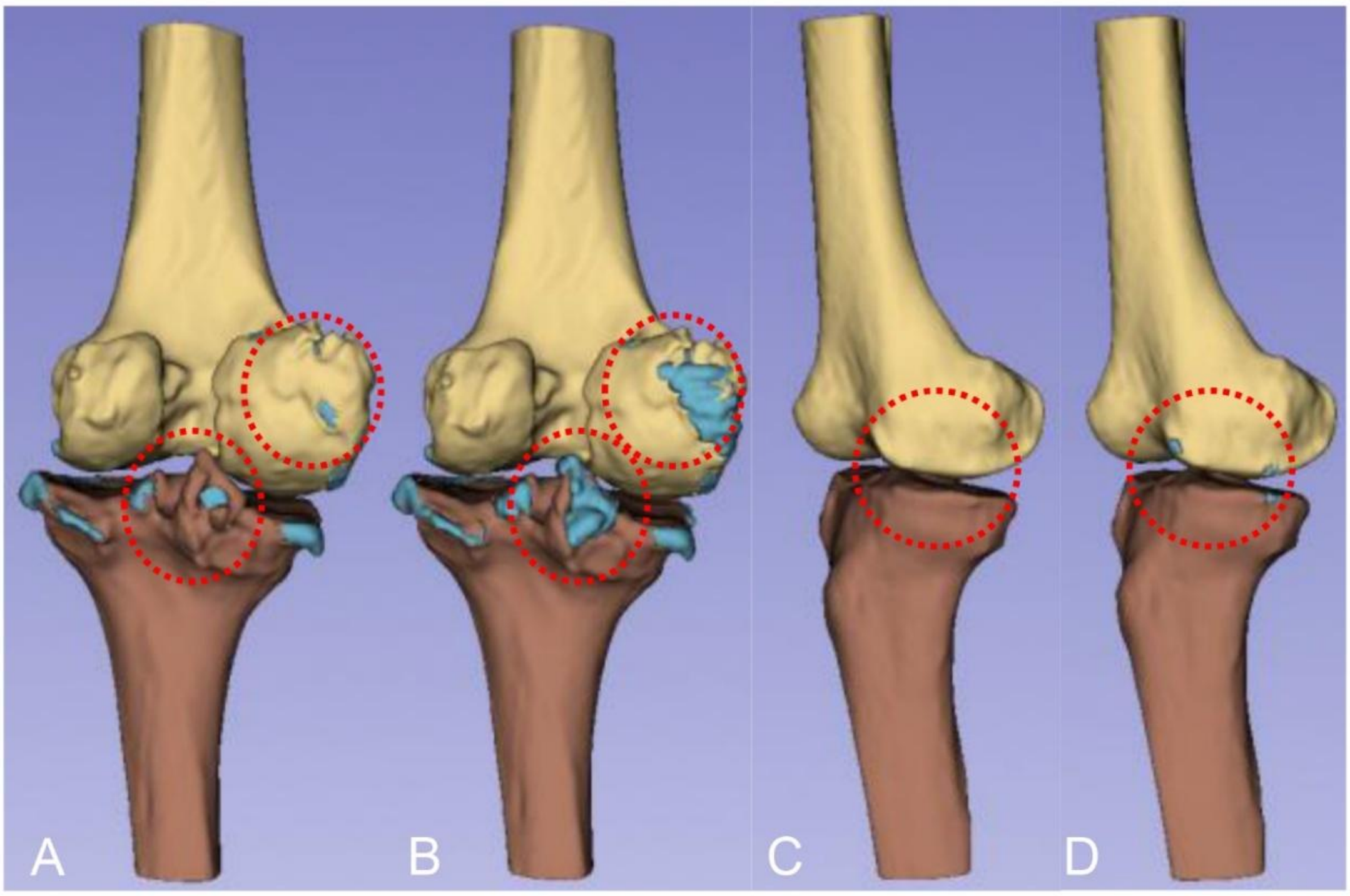

Supplementary Fig. S4. Representative external-validation cases illustrating osteophyte undersegmentation and the effect of very low osteophyte volume on overlap metrics. (A, B) Posterior three-dimensional views of a representative higher-volume knee from external cohort 1 showing automated segmentation and the corresponding blinded expert-consensus annotation, respectively. Cyan denotes osteophytes; the femur and tibia are shown in yellow and brown, respectively. Red dashed circles identify posterior osteophyte regions that were incompletely identified by the automated method, contributing to underestimation of total osteophyte volume (automated, 12.030 cc; expert consensus, 17.497 cc; Dice, 0.815). (C, D) Corresponding views of a representative very-low-volume knee from external cohort 1 showing automated segmentation and blinded expert-consensus annotation, respectively. The red dashed circles indicate the small osteophyte region (automated, 0.0004 cc; expert consensus, 0.068 cc; Dice, 0.013). This example illustrates how a small absolute discrepancy can yield a markedly low overlap metric when osteophyte burden is minimal.

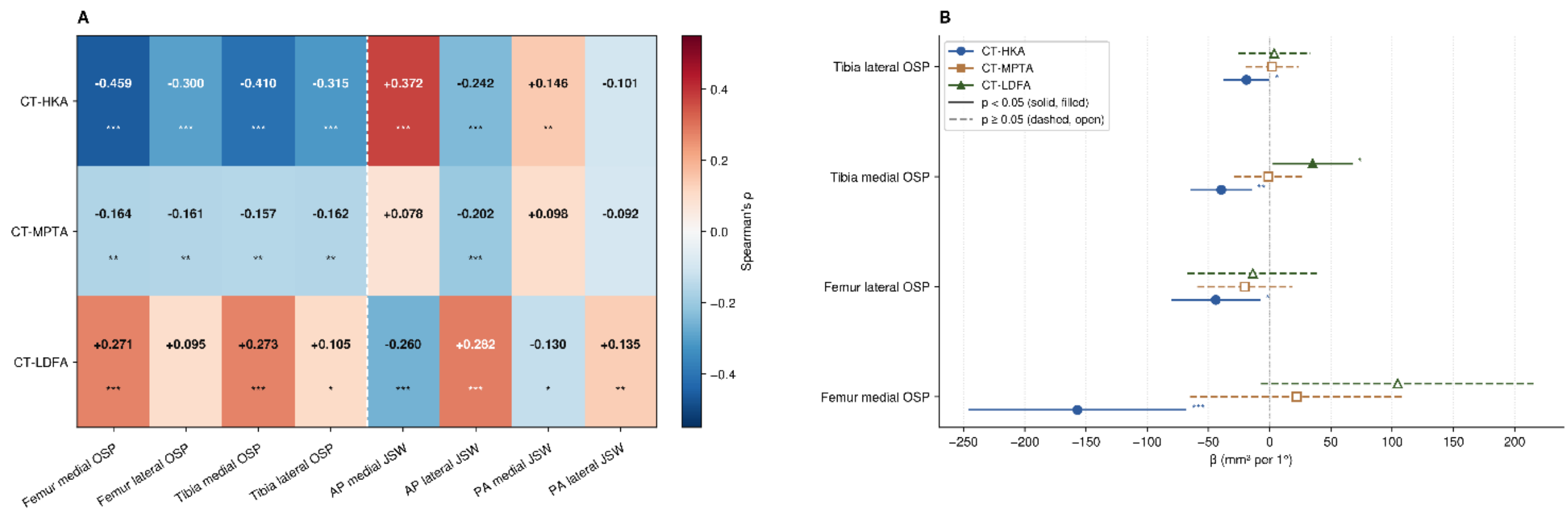

Supplementary Fig. S5. Associations between lower-limb alignment and compartment-specific osteophyte burden (n = 382). (A) Spearman correlations of CT-HKA, CT-MPTA, and CT-LDFA with compartment-specific osteophyte volumes and joint-space-width measurements. Red indicates positive and blue negative correlations; asterisks indicate $p < 0.05$, $p < 0.01$, and $p < 0.001$. (B) Multivariable regression coefficients (β, mm³/degree) and 95% confidence intervals, adjusted for age and sex and estimated using patient-ID cluster-robust standard errors. Solid filled markers indicate $p < 0.05$; dashed open markers indicate $p \geq 0.05$. CT-HKA, CT hip–knee–ankle angle; CT-MPTA, CT medial proximal tibial angle; CT-LDFA, CT lateral distal femoral angle; OSP, osteophyte; JSW, joint-space width.

Supplementary Table S1. CT-derived osteophyte volumes and radiographic joint-space width across Kellgren–Lawrence grades in the exploratory structural analysis cohort (n = 382).

	KL 2 (n=33)	KL 3 (n=238)	KL 4 (n=111)	p	ρ vs KL
**CT osteophyte volume**					

	KL 2 (n=33)	KL 3 (n=238)	KL 4 (n=111)	p	ρ vs KL
Total (mm³)	2,470 ± 2,771	4,043 ± 3,404	5,432 ± 4,108	<0.001	+0.245
Femur medial (mm³)	1,392 ± 1,545	2,208 ± 1,960	2,915 ± 2,094	<0.001	+0.231
Femur lateral (mm³)	536 ± 681	788 ± 771	1,087 ± 1,124	0.001	+0.178
Tibia medial (mm³)	293 ± 478	627 ± 649	850 ± 805	<0.001	+0.218
Tibia lateral (mm³)	250 ± 371	421 ± 506	580 ± 689	0.001	+0.162
**Radiographic JSW**					
AP medial (mm)	4.26 ± 0.81	2.99 ± 0.93	1.40 ± 1.18	<0.001	−0.699
AP lateral (mm)	3.91 ± 0.92	3.98 ± 1.24	3.96 ± 1.41	0.955	+0.011
PA medial (mm)	3.72 ± 2.15	2.23 ± 1.29	1.95 ± 1.56	<0.001	−0.209
PA lateral (mm)	4.12 ± 1.40	3.69 ± 1.38	3.80 ± 1.24	0.189	+0.006

Values are mean ± standard deviation. P values were calculated using the Kruskal–Wallis test. Spearman ρ represents the correlation between each variable and Kellgren–Lawrence grade treated as an ordinal variable. AP, anteroposterior; JSW, joint-space width; KL, Kellgren–Lawrence; OSP, osteophyte; PA, posteroanterior.

Supplementary Table S2. Multivariable associations of CT-based coronal alignment parameters with compartment-specific osteophyte volume in knees with KL grade 2–4.

Outcome and predictor	β (95% CI)	P value
**Femoral lateral osteophyte volume**		
CT-HKA	-44.04 (-79.61 to -8.46)	0.015
CT-MPTA	-20.31 (-59.06 to +18.43)	0.303
CT-LDFA	-13.79 (-67.18 to +39.60)	0.612
Age	-3.68 (-19.52 to +12.16)	0.648

Outcome and predictor	β (95% CI)	P value
Male sex	+207.41 (-33.18 to +448.01)	0.091
**Femoral medial osteophyte volume**		
CT-HKA	-156.94 (-245.13 to -68.75)	<0.001
CT-MPTA	+21.94 (-65.10 to +108.98)	0.620
CT-LDFA	+104.55 (-7.07 to +216.16)	0.066
Age	-1.23 (-34.08 to +31.62)	0.941
Male sex	-109.67 (-545.69 to +326.34)	0.621
**Tibial lateral osteophyte volume**		
CT-HKA	-19.05 (-37.13 to -0.97)	0.039
CT-MPTA	+1.86 (-19.89 to +23.60)	0.867
CT-LDFA	+3.70 (-25.57 to +32.97)	0.804
Age	+2.93 (-6.19 to +12.05)	0.528
Male sex	+29.73 (-124.15 to +183.61)	0.704
**Tibial medial osteophyte volume**		
CT-HKA	-39.55 (-63.64 to -15.45)	0.001
CT-MPTA	-1.19 (-29.27 to +26.90)	0.934
CT-LDFA	+35.09 (+3.26 to +66.93)	0.031
Age	+7.84 (-2.84 to +18.53)	0.150
Male sex	-160.93 (-309.19 to -12.66)	0.033

Models simultaneously included CT-HKA, CT-MPTA, CT-LDFA, age, and sex. Patient-ID cluster-robust standard errors were used to account for correlation between bilateral knees. CT-HKA, CT hip–knee–ankle angle; CT-LDFA, CT lateral distal femoral angle; CT-MPTA, CT medial proximal tibial angle; KL, Kellgren–Lawrence.

Variance inflation factors were 1.34 for CT-HKA, 1.08 for CT-MPTA, and 1.24 for CT-LDFA. Sensitivity analyses using log1p-transformed osteophyte volumes showed materially consistent associations for CT-HKA.
